# Comparing the efficacy of A-PRF and I-PRF in mandibular 3rd molar surgery with similar difficulty index: A comparative, split mouth study

**DOI:** 10.6026/973206300223219

**Published:** 2026-06-30

**Authors:** Prashanth Subhash Pawar, Samir Joshi, Sudhir Pawar, Richa Sharma

**Affiliations:** 1 Department of Oral and Maxillofacial Surgery, Bharati Vidyapeeth (Deemed to be University) Dental College and Hospital, Dhankawadi, Pune, Maharashtra, India;

**Keywords:** Platelet rich plasma, injectable platelet rich Fibrin (I-PRF), advanced-platelet rich fibrin (A-PRF), third molar surgery, similar difficulty index

## Abstract

In regenerative dentistry, platelet rich fibrin (PRF) has been used as a concentrated autologous growth factors capable of encouraging tissue regeneration. Hence, ten patients requiring bilateral mandibular third molar extraction with similar difficulty index were included in the study. Following surgical extraction, A-PRF and I-PRF prepared using low-speed centrifugation protocols were placed into the extraction sockets during separate appointments conducted at a two-week interval. Following surgical extraction, A-PRF and I-PRF prepared using low-speed centrifugation protocols were placed into the extraction sockets during separate appointments conducted at a two-week interval. Thus, A-PRF and I-PRF prepared without anticoagulants using a low-speed centrifugation protocol in mandibular third molar surgery.

## Background:

For more than three decades, platelet concentrations have been used in dentistry as a regenerative material capable of releasing supraphysiological amounts of growth factors that induce tissue regeneration from autologous sources [1]. The variables that influence growth factors, leucocytes and platelets are: Patient's health (blood disease, somatic disease), human factor (patient's lifestyle, health, blood norms) Centrifugation (depending on speed, duration and number of centrifugation spins) and process tubes (empty or with anticoagulation gel) which directly influences the rate of regeneration [2]. Platelets (thrombocytes) have a crucial role in hemostasis; at the same time, their role in regeneration is equally important. The release of growth factors from the platelet's organelle zone occurs after the fibrin clot has formed [3]. The most important growth factors are the following: PDGF (Platelet Derived Growth Factor) which stimulates reparation of tissue, TDGF-β - Transforming growth factor, VEGF - Vascular endothelial growth factor which stimulates angiogenesis, EGF - Epidermal growth factor, IL - Platelet factor interleukin, IGF - Insulin-like growth factor. All these growth factors, platelets and leucocyte will be obtained after centrifuging the blood and simply collecting liquid injectable platelet rich fibrin [I-PRF] into the syringe or in advanced platelet rich fibrin (A-PRF) [4]. Due to the active release of growth factors, the regeneration process is expedited when I-PRF or A-PRF is used. Growth factors are active from the first to second day after I-PRF or A-PRF is applied to injured skin [5]. For 3-4 weeks, active healing with the growth factors is effective. We utilize the original empty tubes without anticoagulants in our practice. Process and benefits A-PRF and I-PRF this aids in the biological coagulation of the blood and the storage of more growth factors for a longer length of time, which facilitates faster tissue repair and regeneration [6]. Platelet rich plasma (PRP) has been widely used in regenerative dentistry, as well as maxillofacial surgery, orthopaedic surgery and cosmetic medicine. Despite this, a number of issues have been note; like the use of anticoagulants such as bovine thrombin and other anticoagulants, which are known to impede tissue regeneration [7]. Platelet rich fibrin (PRF) was created as a first source of autogenous blood-derived growth factors that could be extracted without the use of anti-coagulants, for these reasons. PRF generates a three-dimensional fibrin matrix that also acts as a scaffold for tissue regeneration by functioning as a barrier membrane in guided bone and tissue regeneration (GBR, GTR) treatments while concurrently containing a variety of wound-healing growth factors [8]. PRF has grown in popularity over the last decade and it is now used widely in dentistry and other medical treatments. PRF has been used in dentistry to treat extraction sockets, gingival recessions, palate wound closure, periodontal defect regeneration and hyperplastic gingival tissues. Quicker wound healing, faster angiogenesis, lower expenses and perfect immune-biocompatibility are among the reported benefits [9]. Therefore, it is of interest to report the influence of various patient-related and procedural factors on the quality, composition and regenerative potential of platelet-rich fibrin (PRF) preparations and to evaluate their effectiveness in enhancing tissue healing and clinical outcomes in dentistry.

## Methodology:

Ten patients who are willing to participate were included in the study at Bharati Vidyapeeth Dental College and Hospital, Pune. 10 ml disposable syringe, 24 gauge needle, Duo Centrifuge machine and sterile vacuum plain glass tubes were the important requisites for the study.

## The parameters used for the study were:

[1] Pain (Recorded using the 10 Point Visual Analog Scale), with a score of "0" depicting "no pain" and "10" showing "very severe pain". The pain was evaluated on the 1^st^, 3^rd^ and 7^th^ day post-operatively.

[2] Swelling: It required measuring the distance from the tragus to the soft tissue pogonion on the operated side, measuring the distance from the tragus to the corner of the mouth the on operated side and measuring the distance from the lateral canthus of the eye to the angle of mandible on the operated side. The swelling was also evaluated on 1^st^, 3^rd^ and 7^th^ postoperative day.

[3] Bone Healing: The bone healing of the third molar socket was assessed using OPG. Three parameters, namely, lamina dura score, density score and trabeculae pattern score were assessed. Radiographs were taken immediately after the procedure and on 4^th^ and 8^th^ week postoperatively. Bone healing will was evaluated after 4^th^ and 8^th^ week.

[4] Mouth opening: *i.e.,* measurement of interincisal distance was noted on 1^st^, 2^nd^, 3^rd^ and 7^th^ day using vernier calipers.

## Method of data collection:

Patient requiring bilateral extraction of mandibular 3^rd^ molar teeth reporting to OPD of Department of Oral and Maxillofacial Surgery, Bharati Vidyapeeth Dental College and Hospital, Pune was selected. Informed consent was taken from the patient before the procedure. A detailed case history was taken for all patients participating in the study. Age of the patients ranged from 18-50 years. Healthy subjects (under ASA-1 and 2 classifications) were taken, who required extraction of bilateral mandibular 3^rd^ molar with similar difficulty index. The choice of the quadrant to be operated for study was decided by the toss of a coin by blinded operator. All injections administration and 3^rd^ molar surgery was performed by the same clinician. Also all data was recorded by the same clinician. The time interval between bilateral 3^rd^ molar surgical removal was 2 weeks. All patients were explained about visual analogue proforma pre-operatively. The visual analogue proforma were filled by the patient based on their experiences post-operatively. The study group belonging to Group 1 was administered A-PRF in first appointment whereas the study group belonging to Group 2 were administered I-PRF in 2 weeks interval. On the day of surgical removal 1 hour prior to the procedure 10 ml of blood was withdrawn from patient's cubital fossa with a sterile 10ml syringe.

## Preparation of I-PRF:

10ml of whole blood sample collected without anticoagulant was centrifuged at 700 rpm for 3 minutes at room temperature (DUO CENTRIFUGE. 1ml of I-PRF was collected from upper layer of sample.

## Preparation of A-PRF:

10 ml of whole blood sample collected without anticoagulants using vacuum plain (vacutainer) glass tubes were centrifuged at 3000 rpm for 10 minutes at ambient temperature. Data obtained was entered and sorted in Microsoft Excel (v.2020). Statistical analysis was performed using Statistical package for social sciences (SPSS) software (v.23.0). Descriptive and frequency statistics was performed for all the parameters assessed in the 2 groups. Intergroup comparison for different parameters was assessed using unpaired t-test/independent samples t-test. All statistical tests were performed at 95% confidence intervals; keeping p value of less than 0.05 as statistically significant.

## Results:

Frequency distribution of the study participants according to Bone healing (Lamina dura score) at different time intervals in 2 groups was performed in our study. It was noticed that in A-PRF group, bone healing was less as compared to that of I-PRF group. The lamina dura score in A-PRF group was -2 and -1 whereas in I-PRF group, it was +1 and +2 (Table 7). In our study, Descriptive statistics (Mean + SD) of Pain (VAS scale) at different time intervals in 2 groups was performed. It was found that the mean VAS score was higher in A-PRF group as compared to that of I-PRF group. This mean VAS score reduced as time interval increased for both the groups. At the 7th day, the mean VAS score was lower in I-PRF group as compared to that of A-PRF group. This shows that I-PRF group showed better results as compared to A-PRF (Table 1). Intergroup comparison of time of placement and removal between different groups using Independent samples t-test/Unpaired t-test to assess significant differences. This comparison showed statistically significant differences (p value<0.05) between the groups for time of placement and removal. Intergroup comparison of time of placement and removal between different groups using Independent samples t-test/Unpaired t-test to assess significant differences. This comparison showed statistically significant differences (p value<0.05) between the groups for time of placement and removal. Intergroup comparison of time of placement and removal between different groups using Independent samples t-test/Unpaired t-test to assess significant differences. This comparison showed statistically significant differences (p value<0.05) between the groups for time of placement and removal. Intergroup comparison of time of placement and removal between different groups using Independent samples t-test/Unpaired t-test to assess significant differences. This comparison showed statistically significant differences (p value<0.05) between the groups for time of placement and removal. Intergroup comparison of time of placement and removal between different groups using Independent samples t-test/Unpaired t-test to assess significant differences. This comparison showed statistically significant differences (p value<0.05) between the groups for time of placement and removal. Intergroup comparison of time of placement and removal between different groups using Independent samples t-test/Unpaired t-test to assess significant differences. This comparison showed statistically significant differences (p value<0.05) between the groups for time of placement and removal. Intergroup comparison of time of placement and removal between different groups using Independent samples t-test/Unpaired t-test to assess significant differences. This comparison showed statistically significant differences (p value<0.05) between the groups for time of placement and removal. The present study systematically investigates various clinical outcomes related to pain, swelling, mouth opening and bone healing across two groups, A-PRF and I-PRF, at different time intervals. Table 1 presents the descriptive statistics (Mean ± SD) for pain levels measured using the Visual Analog Scale (VAS) across different time points (1st, 2nd, 3rd and 7th days), highlighting significant differences in pain between the two groups. Swelling measurements at the first day are outlined in Table 2, with parameters such as the distance from the tragus to the soft tissue, the tragus to the corner of the mouth and the lateral canthus of the eye to the angle of the mandible being evaluated. Table 3 extends this analysis to the second day, while Table 4 provides similar descriptive statistics for the third day. The swelling measurements for the seventh day are detailed in Table 5. In addition to swelling, Table 6 offers a thorough evaluation of mouth opening at different time intervals, including the 1st, 2nd, 3rd and 7th days, for both groups. Bone healing progress is also assessed, with Table 7, Table 8 and Table 9 documenting the frequency distribution of participants based on the Lamina Dura, Overall Density and Trabecular Pattern scores at the 4th and 8th weeks. These tables indicate notable variations in bone healing between the two groups, as measured through radiographic scoring. The study further compares the outcomes between the two groups, with Table 10 providing an intergroup comparison of pain scores across the time intervals. Table 11, Table 12, Table 13 and Table 14 present detailed intergroup comparisons of swelling measurements at the first, second, third and seventh days, respectively. Finally, Table 15 highlights the intergroup differences in mouth opening across time intervals and Table 16 evaluates the intergroup differences in bone healing over the 4th and 8th weeks.

## Discussion:

For many clinicians working in the field of regenerative dentistry, the use of barrier membranes, bone grafting materials and bioactive growth factors to aid fresh tissue regeneration has become routine. Platelet concentrates, such as A-PRF and I-PRF; both employ supra-physiological quantities of autologous growth factors obtained from the patient's blood and are capable of expediting soft and hard tissue regeneration [10]. Because of which it is gaining lit of clinical important in oral surgery. In 2001, platelet-rich-fibrin (PRF) was introduced as the initial step toward the development of blood-derived PRF matrices that do not require extra anticoagulants. The stimulation of the physiological coagulation cascade by centrifugation within a specialized glass-based tube results in the production of a fibrin clot enriched with cells from the peripheral blood, such as platelets and leukocytes [11]. One of the most notable outcomes of this study is that altering the procedure in terms of reduced duration and speed of centrifuge results in a distinct neutrophil granulocyte dispersion pattern. Furthermore, platelets and the fibrin network are recognized to play an important role in wound healing. Leukocytes participate in angiogenesis and lymph genesis, whereas the fibrin network is a key player in the early stages of wound healing due to its synergistic effects with platelets and its function as a cytokine reservoir [12]. The use of large relative centrifugal forces (RCF) (708 g) throughout the centrifugation process was stated as necessary to create PRF. Platelets and inflammatory cells inside the fibrin scaffold aggregate mostly in the proximal section of the PRF clot, according to prior histological examinations of the PRF clot. Fine-tuning the centrifugation process in terms of RPM and duration results in the formation of injectable PRF (I-PRF) with increased platelet and leukocyte counts, particularly neutrophil granulocytes dispersed equally throughout I-PRF. as compared to advanced PRF (A-PRF) [13]. IPRF released more growth factors as compared to A-PRF during healing phase because I-PRF preserves more growth factor releasing cells as compared to A-PRF. I-PRF and A-PRF experienced a controlled and long-term release of growth factors, as established in this study. While it has been demonstrated that I-PRF includes more live progenitor cells and platelets than A-PRF, the large increase in total protein release that follows may provide further therapeutic benefits. Growth factors, we reasoned, may be administered to stimulate wound healing, reduce post-operative problems including pain, edema and trismus (interincisal distance) and improve patient outcomes as confirmed in the study [14]. The PRF scaffold concept appears to be an excellent source of healing components. A scaffold made from autologous blood can be a one-of-a-kind source of hematopoietic stem cells (HSCs), which are critical in regenerative medicine. Many studies in the last decade have shown HSCs' tremendous differentiation capability [15]. Furthermore, with PRF has greater cytokine inclusion in the fibrin mesh (intrinsic cytokines). Because of which these cytokines will only be produced and utilized during the early cicatricle matrix remodeling, this arrangement indicates that they will have a longer lifetime (long-term effect). When the cells start cicatricle matrix remodeling, *i.e.,* when they need to be stimulated to start wounded site rebuilding, the cytokines are accessible *in situ* for a suitable duration [16]. The regeneration process is expedited after surgical removal of the mandibular 3rd molar with IPRF or APRF due to the active release of growth factors. Growth factors become active 7 to 14 days after application of I-PRF or A-PRF into the extraction socket. For 3-4 weeks, active healing with the growth factors is effective. I-PRF has an advantage over A-PRF in that it releases growth factors earlier and stays longer than A-PRF, resulting in less discomfort, less edema, expediated bone repair and a return to normal mouth opening in a short period of time [17]. Based on result of the study, we concluded that I-PRF is more successful than A-PRF as clot develops a robust complex 3-dimensional architecture.

## Conclusion:

I-PRF showed significantly superior clinical and radiographic outcomes compared to A-PRF following bilateral mandibular third molar surgery. Patients treated with I-PRF showed reduced postoperative pain and swelling, improved mouth opening and enhanced bone healing at different postoperative intervals. Thus, the low-speed centrifugation protocol used for I-PRF preparation may contribute to improved regenerative potential through greater preservation of platelets, leukocytes and growth factors.

## Figures and Tables

**Table 1:** Descriptive statistics (Mean ± SD) of Pain (VAS scale) at different time intervals in 2 groups

**Parameter**	**Groups**	**N**	**Minimum**	**Maximum**	**Mean**	**Std. Deviation**
Pain 1st day	A-PRF	10	4	5	4.7	0.48305
	I-PRF	10	3	4	3.6	0.5164
Pain 2nd day	A-PRF	10	3	4	3.5	0.52705
	I-PRF	10	2	3	2.2	0.42164
Pain 3rd day	A-PRF	10	1	3	2	0.66667
	I-PRF	10	0	1	0.9	0.31623
Pain 7th day	A-PRF	10	0	1	0.8	0.42164
	I-PRF	10	0	1	0.1	0.31623

**Table 2:** Descriptive statistics (Mean ± SD) of swelling at different measurements at 1st day in 2 groups

**Parameter**	**Groups**	**N**	**Minimum**	**Maximum**	**Mean**	**Std. Deviation**
Distance from the tragus to the soft tissue	A-PRF	10	11.4	12.8	12.02	0.42895
	I-PRF	10	10.6	11.6	11.17	0.3335
Distance from tragus to the corner of the mouth	A-PRF	10	11.8	14.4	13.16	0.76333
	I-PRF	10	11	12.8	11.95	0.56814
Distance from lateral canthus of the eye to angle of mandible	A-PRF	10	11.6	12.9	12.51	0.38137
	I-PRF	10	11.2	12.2	11.83	0.3199

**Table 3:** Descriptive statistics (Mean ± SD) of swelling at different measurements at 2nd day in 2 groups

**Parameter**	**Groups**	**N**	**Minimum**	**Maximum**	**Mean**	**Std. Deviation**
Distance from the tragus to the soft tissue	A-PRF	10	11	13.2	12.26	0.54813
	I-PRF	10	10.6	12.8	11.73	0.60745
Distance from tragus to the corner of the mouth	A-PRF	10	12	15.4	13.79	1.20411
	I-PRF	10	11.5	13.4	12.45	0.7261
Distance from lateral canthus of the eye to angle of mandible	A-PRF	10	11.8	13.2	12.55	0.42753
	I-PRF	10	10.9	12.1	11.53	0.42177

**Table 4:** Descriptive statistics (Mean±SD) of swelling at different measurements at 3rd day in 2 groups

**Parameter**	**Groups**	**N**	**Minimum**	**Maximum**	**Mean**	**Std. Deviation**
Distance from the tragus to the soft tissue	A-PRF	10	11.1	13.3	12.12	0.73151
	I-PRF	10	10.6	12.1	11.51	0.45814
Distance from tragus to the corner of the mouth	A-PRF	10	12.5	14.9	13.39	0.65904
	I-PRF	10	11.4	13.4	12.33	0.67338
Distance from lateral canthus of the eye to angle of mandible	A-PRF	10	10.8	13.5	12.11	1.04398
	I-PRF	10	10	11.8	11.16	0.53375

**Table 5:** Descriptive statistics (Mean + SD) of swelling at different measurements at 7th day in 2 groups

**Parameter**	**Groups**	**N**	**Minimum**	**Maximum**	**Mean**	**Std. Deviation**
Distance from the tragus to the soft tissue	A-PRF	10	11.4	12.6	11.88	0.4492
	I-PRF	10	9.6	10.8	10.23	0.39455
Distance from tragus to the corner of the mouth	A-PRF	10	12	14.7	13.26	0.65693
	I-PRF	10	10.2	11.6	10.63	0.38601
Distance from lateral canthus of the eye to angle of mandible	A-PRF	10	11.8	12.6	12.17	0.29078
	I-PRF	10	10	11.2	10.71	0.34785

**Table 6:** Descriptive statistics (Mean±SD) of mouth opening at different time intervals in 2 groups

**Parameter**	**Groups**	**N**	**Minimum**	**Maximum**	**Mean**	**Std. Deviation**
Mouth opening 1st day	A-PRF	10	23.4	27.6	25.38	1.36772
	I-PRF	10	25.8	29.2	27.76	1.10775
Mouth opening 2nd day	A-PRF	10	22.4	29.8	26.54	2.15623
	I-PRF	10	27.8	30.8	29.43	1.04992
Mouth opening 3rd day	A-PRF	10	22.6	36.5	29.08	3.67236
	I-PRF	10	28.9	38.5	32.44	3.29215
Mouth opening 7th day	A-PRF	10	29.6	37.5	33.33	2.66127
	I-PRF	10	32.4	39.6	36.57	2.44224

**Table 7:** Frequency distribution of the study participants according to Bone healing (Lamina dura score) at different time intervals in 2 groups

**Bone healing**	**4th week**		**8th week**		**Bone healing**	**4th week**		**8th week**	
A-PRF	Frequency (n)	Percent (%)	Frequency (n)	Percent (%)	I-PRF	Frequency (n)	Percent (%)	Frequency (n)	Percent (%)
-1	1	10	9	90	1	6	60	-	-
-2	9	90	1	10	2	4	40	10	100
Total	10	100	10	100	Total	10	100	10	100

**Table 8:** Frequency distribution of the study participants according to Bone healing (Overall density score) at different time intervals in 2 groups

**Bone healing**	**4th week**		**8th week**		**Bone healing**	**4th week**		**8th week**	
A-PRF	Frequency (n)	Percent (%)	Frequency (n)	Percent (%)	I-PRF	Frequency (n)	Percent (%)	Frequency (n)	Percent (%)
-1	2	20	8	80	1	2	20	-	-
-2	8	80	2	20	2	8	80	10	100
Total	10	100	10	100	Total	10	100	10	100

**Table 9:** Frequency distribution of the study participants according to Bone healing (Trabecular pattern score) at different time intervals in 2 groups

**Bone healing**	**4th week**		**8th week**		**Bone healing**	**4th week**		**8th week**	
A-PRF	Frequency (n)	Percent (%)	Frequency (n)	Percent (%)	I-PRF	Frequency (n)	Percent (%)	Frequency (n)	Percent (%)
-1	2	20	6	60	1	4	40	-	-
-2	8	80	4	40	2	6	60	10	100
Total	10	100	10	100	Total	10	100	10	100

**Table 10:** Intergroup comparison of Pain between 2 groups at different time intervals

**Parameter**	**Groups**	**Mean**	**t value**	**df**	**P value**
Pain 1st day	A-PRF	4.7	4.919	18	.000*
	I-PRF	3.6			
Pain 2nd day	A-PRF	3.5	6.091	18	.000*
	I-PRF	2.2			
Pain 3rd day	A-PRF	2	4.714	18	.000*
	I-PRF	0.9			
Pain 7th day	A-PRF	0.8	4.2	18	.001*
	I-PRF	0.1			
*p value <0.05 statistically significant

**Table 11:** Intergroup comparison of swelling measurements between 2 groups at 1^st^ day

**Parameter**	**Groups**	**Mean**	**t value**	**df**	**P value**
Distance from the tragus to the soft tissue	A-PRF	12.02	4.947	18	.000*
	I-PRF	11.17			
Distance from tragus to the corner of the mouth	A-PRF	13.16	4.021	18	.001*
	I-PRF	11.95			
Distance from lateral canthus of the eye to angle of mandible	A-PRF	12.51	4.32	18	.000*
	I-PRF	11.83			
*p value <0.05 statistically significant

**Table 12:** Intergroup comparison of swelling measurements between 2 groups at 2^nd^ day

**Parameter**	**Groups**	**Mean**	**t value**	**df**	**P value**
Distance from the tragus to the soft tissue	A-PRF	12.26	2.048	18	0.055
	I-PRF	11.73			
Distance from tragus to the corner of the mouth	A-PRF	13.79	3.014	18	0.007
	I-PRF	12.45			
Distance from lateral canthus of the eye to angle of mandible	A-PRF	12.55	5.371	18	0
	I-PRF	11.53		
*p value <0.05 statistically significant

**Table 13:** Intergroup comparison of swelling measurements between 2 groups at 3^rd^ day

**Parameter**	**Groups**	**Mean**	**t value**	**df**	**P value**
Distance from the tragus to the soft tissue	A-PRF	12.12	2.235	18	.038*
	I-PRF	11.51			
Distance from tragus to the corner of the mouth	A-PRF	13.39	3.558	18	.002*
	I-PRF	12.33			
Distance from lateral canthus of the eye to angle of mandible	A-PRF	12.11	2.562	18	.020*
	I-PRF	11.16			
*p value <0.05 statistically significant

**Table 14:** Intergroup comparison of swelling measurements between 2 groups at 7^th^ day

**Parameter**	**Groups**	**Mean**	**t value **	**df**	**P value**
Distance from the tragus to the soft tissue	A-PRF	11.88	8.727	18	.000*
	I-PRF	10.23			
Distance from tragus to the corner of the mouth	A-PRF	13.26	10.915	18	.000*
	I-PRF	10.63			
Distance from lateral canthus of the eye to angle of mandible	A-PRF	12.17	10.183	18	.000*
	I-PRF	10.71			
*p value <0.05 statistically significant

**Table 15:** Intergroup comparison of mouth opening between 2 groups at different time intervals

**Parameter**	**Groups**	**Mean**	**t value**	**df**	**P value**
Mouth opening 1st day	A-PRF	25.38	-4.276	18	0.000*
	I-PRF	27.76			
Mouth opening 2nd day	A-PRF	26.54	-3.811	18	0.001*
	I-PRF	29.43			
Mouth opening 3rd day	A-PRF	29.08	-2.154	18	0.045*
	I-PRF	32.44			
Mouth opening 7th day	A-PRF	33.33	-2.837	18	0.011*
	I-PRF	36.57			
*p value <0.05 statistically significant

**Table 16:** Intergroup comparison of bone healing between 2 groups at different time intervals

**Parameter**	**Groups**	**Mean**	**t value**	**df**	**P value**
Bone healing 4th week LD	A-PRF	1.1	-1.567	18	0.135
	I-PRF	1.4			
Bone healing 4th week OD	A-PRF	1.3	-4.583	18	0.000*
	I-PRF	2			
Bone healing 4th week TB	A-PRF	1.2	-3.182	18	0.005*
	I-PRF	1.8			
Bone healing 8th week LD	A-PRF	1.5	-2.058	18	0.05*
	I-PRF	1.9			
Bone healing 8th week OD	A-PRF	1.1	-9	18	0.000*
	I-PRF	2			
Bone healing 8th week TB	A-PRF	1.4	-2.611	18	0.018
	I-PRF	1.9			
*p value <0.05
statistically significant
